# Bone Marrow Mesenchymal Stem Cells in Acute-on-Chronic Liver Failure Grades 2 and 3: A Phase I-II Randomized Clinical Trial

**DOI:** 10.1155/2021/3662776

**Published:** 2021-08-04

**Authors:** Fernando Comunello Schacher, Annelise Martins Pezzi da Silva, Lucia Mariano da Rocha Silla, Mario Reis Álvares-da-Silva

**Affiliations:** ^1^Graduate Program Sciences in Gastroenterology and Hepatology, School of Medicine, Universidade Federal do Rio Grande do Sul, Porto Alegre, Brazil; ^2^Hematology Division, Hospital de Clínicas de Porto Alegre, Porto Alegre, Brazil; ^3^GI/Liver Division, Hospital de Clínicas de Porto Alegre, Porto Alegre, Brazil

## Abstract

**Introduction:**

Acute-on-chronic liver failure (ACLF) is an acute liver decompensation in cirrhotic patients, which leads to organ failures and high short-term mortality. The treatment is based on the management of complications and, in severe cases, liver transplantation. Since specific treatment is unavailable, we aimed to evaluate the safety and initial efficacy of bone marrow mesenchymal stem cells (BM-MSC) in patients with ACLF Grades 2 and 3, a population excluded from previous clinical trials.

**Methods:**

This is a randomized placebo-controlled phase I-II single center study, which enrolled 9 cirrhotic patients from 2018 to 2020, regardless of the etiology. The control group (*n* = 5) was treated with standard medical therapy (SMT) and placebo infusion of saline. The intervention group (*n* = 4) received SMT plus 5 infusions of 1 × 10^6^ cells/kg of BM-MSC for 3 weeks. Both groups were monitored for 90 days. A Chi-square test was used for qualitative variables, and the *t*-test and Mann–Whitney *U* test for quantitative variables. The Kaplan–Meier estimator was used to build survival curves. In this study, we followed the intention-to-treat analysis, with a significance of 5%.

**Results:**

Nine patients with a mean Child–Pugh (CP) of 12.3, MELD of 38.4, and CLIF-C score of 50.7 were recruited. Hepatitis C and alcohol were the main etiologies. The average infusion per patient was 2.9 and only 3 patients (2 in control and 1 in the BM-MSC group) received all the protocol infusions. There were no infusion-related side effects, although one patient in the intervention group presented hypernatremia and a gastric ulcer, after the third and fifth infusions, respectively. The survival rate after 90 days was 20% (1/5) for placebo versus 25% (1/4) for the BM-MSC. The patient who completed the entire MSC protocol showed a significant improvement in CP (C-14 to B-9), MELD (32 to 22), and ACLF (grade 3 to 0).

**Conclusion:**

BM-MSC infusion is safe and feasible in patients with ACLF Grades 2 and 3.

## 1. Introduction

Acute-on-chronic liver failure (ACLF) is a syndrome in patients with chronic liver disease, which is characterized by acute decompensation, organ failure, and high short-term mortality [1]. Since the first definition of ACLF in 2002 [2], there have been several attempts to put forward a better definition and diagnostic criteria by some Hepatology societies worldwide [3–6]. However, the methodology used was inappropriate, which has led to a lack of consensus.

In 2013, after the publication of a prospective multicenter observational study, the CANONIC trial, it was possible to define this syndrome in a more detailed and accurate way. Diagnostic criteria used in these cohorts applied a new scoring system, the Chronic Liver Failure Sequential Organ Failure Assessment score (CLIF-SOFA), improving the standardization of diagnostic criteria [7].

Despite the progress in the characterization and the diagnosis of ACLF, its management is still limited, which explains the high short-term mortality. Currently, therapeutic alternatives include treating the underlying etiology when feasible, controlling the precipitating factors, and supportive measures for organ failures. In cases of clinical deterioration, a liver transplantation (LT) is an option [8, 9]. However, due to the scarcity of donors, the severity of the organ failures, and the risk of being futile, LT is restricted to a few cases.

In this context, other strategies have been studied, including extracorporeal liver support systems [10, 11], immunomodulatory treatments such as granulocyte colony-stimulating factor [12, 13], and faecal microbiota transplantation [14]. One of the most promising treatments is the use of mesenchymal stem cells (MSC), which has been shown to have anti-inflammatory effects, reducing both hepatocyte damage [15] and hepatic stellate cell activation [16]. Although few trials have shown benefits from its use [17–20], and given that its role in ACLF is still uncertain, the lack of effective treatment has led to an increased interest in exploring MSC therapy further for this condition.

The aim of this study was to evaluate the security and eventual efficacy of bone-marrow mesenchymal stem cell (BM-MSC) transfusions in patients with ACLF.

## 2. Methods

### 2.1. Study Design, Criteria, and Ethical Issue

This was a double blind, placebo-controlled, Phases I and II, randomized clinical trial carried out in one center in Brasil (Hospital de Clínicas de Porto Alegre) from September 2018 to January 2020. The purpose was to evaluate the safety and efficacy of allogeneic bone-marrow mesenchymal stem cells (BM-MSC) infusion in patients with ACLF Grades 2 and 3.

We used the ACLF definitions based on the CANONIC trial [7] in patients with a previously known history of cirrhosis, who were hospitalized due to acute decompensation of the liver, brought about by conditions such as voluminous ascites, hepatic encephalopathy, gastrointestinal hemorrhage, bacterial infection, or any combination of these. Inclusion criteria also required (a) fulfilling ACLF diagnostic criteria and ACLF Grade 2 or 3 and (b) being aged between 18 and 70 years. Exclusion criteria were (a) patient's or family member's refusal; (b) hepatocellular carcinoma (HCC); (c) formal contraindication for liver transplantation (e.g., advanced heart or pulmonary disease); (d) pregnancy and lactation; (e) previous liver transplantation; (f) HIV coinfection; (g) ACLF grade 1; (h) patients admitted for elective procedures; and (i) renal chronic disease requiring dialysis.

The study protocol was approved by the Ethics Committee of the Hospital de Clínicas de Porto Alegre (register number: 92330718.0.0000.5327) and by the Brazilian Registry of Clinical Trials (register number: RBR-8n8csf).

### 2.2. Randomization and Masking

Before the start of the trial, manual randomization was performed, prespecifying which patient would receive BM-MSC or placebo. Both the medical team that assisted the patient and the patient were unaware of the assignment group.

### 2.3. Patients and Procedures

Nine patients were eligible and either the patients or a family member gave a signed informed consent when the patient was not able to sign it. A physician trained and certified for the interview and collection of the informed consent was also responsible for the collection of clinical and biochemical data from electronic medical records. After this, patients were assigned standard medical treatment (SMT) with allogeneic bone-marrow mesenchymal stem cells or SMT plus placebo.

In the intervention group, MSC was given in the form of 5 IV infusions of 1.0 × 10^6^ cells/kg, twice a week for 2 weeks and one dose in the third week; the placebo group received, in a similar recipient, the same amount of saline.

Vital signs and clinical status were documented immediately before and up to one hour after the end of the infusion. All possible adverse reactions (e.g., rash, fever, and changes in blood pressure) were recorded every 30 minutes. One day after infusion, clinical status and possible adverse reactions (e.g., diarrhea) were reassessed. Laboratory tests along with evaluation of the Child–Pugh (CP), Model for End-Stage Liver Disease (MELD), MELD-Na, and CLIF-SOFA scores were performed before the first infusion and at 28 and 90 days following treatment.

### 2.4. Allogeneic BM-MSCs

The mesenchymal cells were obtained from a bag and filter from bone marrow donors at the Hospital de Clínicas de Porto Alegre, used in hematopoietic stem cell transplantation, after the consent had been signed. Such procedures have no influence on this type of transplantation and studies have previously shown that it is possible to obtain these cells for cell therapy, with the advantage that this material aggregates the marrow donor serology data [21].

#### 2.4.1. MSC Cultivation

The bags and filters used in the hematopoietic stem cell transplantation that served as a source of MSC for cultivation were sent to the Advanced Cell Processing Center at the Hospital de Clínicas de Porto Alegre shortly after transplant. The cells were removed by eluting the filter and bag with saline. After isolation by centrifugation, the cells were counted in the Neubauer chamber, and the viability was verified by the exclusion method with Tripan Blue dye. The cells were then plated in culture flasks at a density of 300,000 live nucleated cells/cm^2^ in DMEM medium (Eagle medium modified by Dulbecco, Gibco) supplemented with 10% human platelet lysate and with 1% penicillin/streptomycin antibiotic added (Gibco). The culture flasks were then transferred to incubators humidified with 5% CO_2_, at 37°C. Cell growth was monitored through microscopy, and when a confluence of approximately 80% was reached, the cells were detached from the flask using 0.05% trypsin/EDTA (Invitrogen) and plated in new culture bottles at a concentration of 5,000 cells/cm^2^. The cells were expanded until the second passage (P2), at which time they were cryopreserved and stored at −80°C while awaiting quality control tests.

#### 2.4.2. Platelet Lysate

Antibodies against Bovine Fetal Serum (BFS) proteins were detected in patients who received MSC expanded with this supplement [22]. As a substitute for BFS, human serum has been used successfully through platelet lysate (LP). In vitro studies have shown LP to be as effective as BFS for the expansion of MSC [22, 23], and another study showed an expansion of mesenchymal stem cells cultured with LP 3.75 times greater than BFS [21]. Therefore, LP is safer from a biological point of view and is at least as efficient as BFS for cell expansion.

#### 2.4.3. Cryopreservation

The cells were cryopreserved in a transfer bag with cell counts equivalent to 1 × 10^6^ live cells per kg, in a volume of 25 ml composed of 17.5 ml of albumin +5 ml of 6% hydroxyethyl starch (HES) + 2.5 ml of dimethylsulfoxide (DMSO). They were stored at −80°C and quarantined until quality control tests were ready for clinical use.

#### 2.4.4. Quality Control

*(1) Immunophenotyping*. The cells were analyzed for their membrane markers by flow cytometry on a FACSCantoII cytometer (BD Biosciences) at the time of cryopreservation and immediately before being administered to the patient. CTMs must express the markers DC73, DC90, and DC105 and must be negative for DC14, DC34, DC19, HLADR, and DC45 [24]. Harvested cells were adjusted for cell concentration of 1 × 10^6^/ml in PBS, and at 100 *μ*l the suspension per tube was incubated with the respective monoclonal antibodies for 30 minutes at room temperature and protected from light. After being washed, the cells were then fixed with 4% paraformaldehyde and analyzed on the flow cytometer.

The data acquisition and analysis of the sample was performed using Diva software on a FACSCantoII flow cytometer (BD Biosciences). The results were evaluated using a dot-plot graph and histogram, and the fluorescence intensity was measured using the MFI (mean of fluorescence intensity) [24, 25].

*(2) Differentiation Tests*. MSCs must have the ability to differentiate in osteocyte, adipocyte, and chondrocyte strains. At the P2 passage, three small aliquots were differentiated in each of the three cell lines using specific commercial reagents as per the manufacturer's instructions (StemPro®, Gibco) and were recorded by microscopy.

*(3) Test to Check for the Presence of Mycoplasma*. The presence of mycoplasma was tested with a commercial kit (VenorGeM Mycoplasma Detection Kit, Sigma-Aldrich). This kit uses the polymerase chain reaction (PCR), established as the gold standard, due to its greater sensitivity in detecting *Mycoplasma* contamination in cell cultures. This kit is able to detect 1–5 fg of contaminating DNA in 2–5 units of *Mycoplasma* per sample volume. The primer set is specific to a highly conserved region, or more precisely, the 16S rRNA coding region in the Mycoplasma genome. This allows for the detection of all *Mycoplasma* species tested so far, which are normally found as contaminants in cell cultures.

*(4) Test to Check for the Presence of Endotoxin*. To detect endotoxins in the sample, an Endosafe®-PTS test (Charles River, USA) was used with cartridges with sensitivity of 0.05–0.1 EU/ml. The cartridge has 4 channels, two positive controls and two for reading the sample. The presence of endotoxins in the sample triggers an enzymatic reaction, generating a yellow color, through the cleavage of a chromogenic substrate. These kinetics are read by the optical cells of the PTS-Endosafe equipment.

*(5) Sterility*. To check for the presence of microorganisms in the cell product, the supernatant (final product) from a 3 ml aliquot culture was subjected to aerobic and anaerobic blood culture tests carried out (by means of the automated BactAlert® system) at the Microbiology Laboratory of the Hospital de Clínicas de Porto Alegre.

### 2.5. Thawing and Preparing Cells for Infusion

For infusion in a patient, the transfer bag was thawed in a water bath at 37°C for 2 minutes and in a closed system: 15 mL of albumin (20%), 3.75 mL of anticoagulant solution for apheresis (ACD), and 56.25 mL of saline solution (0.9%) were added for dilution of DMSO. An aliquot was then removed for counting and cell viability. Preferably 1 × 10^6^ live cells/kg were infused and only bags with viability above 70% were used. MSCs were administered intravenously to patients who met the criteria for inclusion in this study.

### 2.6. MSC Infusions

We aimed to perform five infusions of MSC in a peripheral vein in a dose of 1 × 10^6^ cells/kg over three weeks: two in the first week, two in the second week, and one in the third week. The MSCs were infused in a transfer bag using equipment with a macroaggregated retention filter with a minimum duration of 10 to 20 minutes. Throughout the procedure and 60 minutes after the end of the infusion, vital signs were recorded every 30 minutes. The presence of transfusion reactions and the patient's clinical evolution were also recorded.

### 2.7. Outcomes

The primary outcomes were incidence of adverse events and survival after infusions. Secondary outcomes were liver transplantation rates and changes in CP, MELD, MELD-Na, and CLIF-SOFA scores.

We also evaluated liver function through bilirubin, prothrombin time and albumin levels as well as inflammatory stage via leukocytes and C-reactive protein after the first infusion of MSC.

### 2.8. Sample Size and Statistical Analysis

As there are few studies analyzing the use of BM-MSC, we defined a convenience sample of 9 patients, four in the intervention group and five in the placebo group.

For quantitative variables, both the mean and the standard deviation were presented, while for the qualitative variables, the absolute frequency was followed by the percentage that this represents of the total (*n* (%)).

For the comparison of groups, a Chi-square test was used for qualitative variables, and the *t*-test for quantitative variables, and, when abnormal, the Mann–Whitney *U* test. The Kaplan–Meier estimator was used to estimate survival curves and the curves were compared using the log-rank test. We followed the intention-to-treat analysis, which was performed using a software R, version 3.6.0. Tests were performed at 5% significance level.

## 3. Results

### 3.1. Population and General Characteristics at Baseline

Of the 9 study participants, four received BM-MSC. There were no significant differences in baseline clinical and biochemical profiles between the two groups (Tables 1 and 2). The mean age was 54.4 years and 66.7% were male. Two-thirds of patients had alcohol and hepatitis C as the etiology of cirrhosis and the main reason for development of ACLF was infection (44.4%). The mean scores were CP 12.3 (SD ± 1.2), MELD 38.4 (SD ± 7.3), ACLF grade 2.3 (SD ± 0.5), and CLIF-C 50.7 (SD ± 10.9). Six patients died before the end of the study protocol (3 in the placebo group and 3 in the intervention group).

### 3.2. Safety

There were no adverse events observed up to 1-hour after infusion. The most important adverse events were hypernatremia (162 mEq/L) and gastrointestinal bleeding due to a gastric ulcer, which was observed after the third and fifth doses, respectively. Both adverse reactions were found in the same patient from the MSC group and were not related to MSC infusions.

### 3.3. Efficacy

The average number of infusions in all patients was 2.9, with a median of 2, with at least 1 and a maximum of 5 infusions. Only three patients received all the five doses according to the study protocol, two in the placebo group and one in the intervention group.

**Figure 1:**
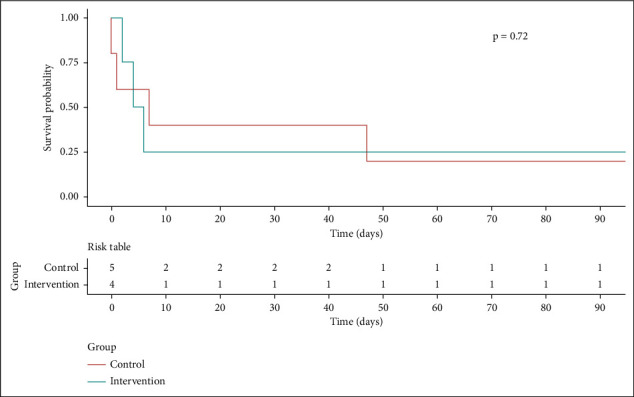
Kaplan–Meier survival curves (*n* = 9).

The median survival time was 32 days with a standard deviation of 47.4 days. The survival rate after 90 days was 20% for the placebo group (1/5) and 25% for the MSC group (1/4) (Figure 1). On day 28 of the follow-up, six patients had died, and of the three who remained alive, two were from the placebo group. At 90 days after the infusions, two patients remained alive (one in the placebo and one in the MSC groups). None of the patients received a liver transplant over the period of the study.

**Figure 2:**
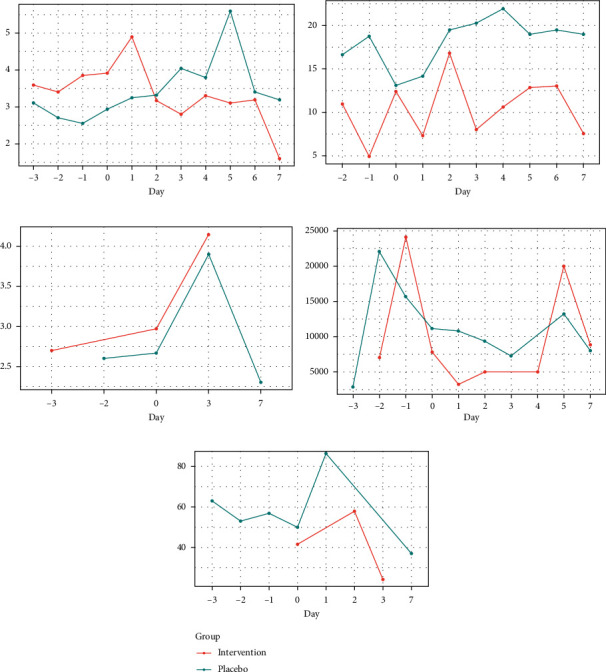
Changes in liver and inflammatory laboratory tests from three days before the first dose of MSC through the seventh day after the first infusion. PT, prothrombin time; TB, total bilirubin; Alb, albumin; CRP, C-reactive protein. (a) PT evolution. (b) TB evolution. (c) Alb evolution. (d) Leukocytes evolution. (e) CRP evolution.

Additionally, we analyzed the liver and inflammatory laboratory tests from three days before the first dose of MSC through to the seventh day after the first infusion. In the intervention group, there was a slight decrease in prothrombin time and total bilirubin and a small increase in albumin levels, features which were not observed in the placebo group. As for inflammatory features, C-reactive protein, and leukocytes, there were no differences between the groups (Figure 2).

**Figure 3:**
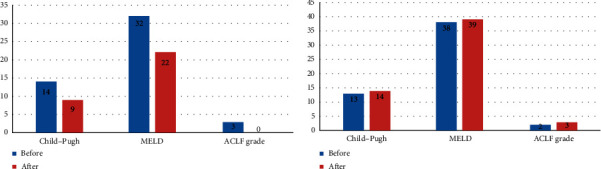
Variations regarding Child–Pugh, MELD and ACLF scores before and after the infusion of 5 doses of the intervention group ((a); *n* = 1) and in the placebo group after 90 days ((b); *n* = 2). MELD, Model for end-stage liver disease; ACLF, acute-on-chronic liver failure.

Regarding those patients who survived until the end of the protocol, the patient in the MSC had a clear improvement in the liver function (Figure 3), which was not observed in the placebo group.

## 4. Discussion

Given that there is no approved specific therapy for ACLF, our study aimed to evaluate the safety and efficacy of BM-MSC infusions in patients with Grades 2 and 3 ACLF. Although we have demonstrated the safety of MSC in this population, 90-day survival rates were similar between the MSC and placebo groups. As far as we have been able to determine, this is the first MSC trial under such severe forms of ACLF as well as the first to enroll patients with liver disease of a different etiology.

Unlike the other trials which have evaluated MSC in ACLF using the Asian Pacific Association for the Study of the Liver (APASL) criteria for defining ACLF [17–20], we decided to employ a different criterion, the EASL-CLIF, because there is growing evidence which shows that it performs better in defining ACLF [26–28]. This makes comparing our data with those of previous studies somewhat complicated. In addition to the criteria used for enrollment, there are many other differences between the previous protocols of infusion of multipotent cells in ACLF and ours. First of all, they include only patients infected with hepatitis B (HBV). Moreover, not all were cirrhotic. Indeed, these studies probably included chronic hepatitis patients with HBV flares, as they had high alanine transaminase levels and high viral loads as well. Therefore, the overall results obtained were not only from the use of MSC but from the association of MSC and antivirals as well. Additionally, MSC origin and infusion protocols were not the same utilized in the present study. Most studies administrated MSC from umbilical cord [17, 19, 20]. Lin et al. [18] also treated patients with BM-MSC in a different protocol (1 infusion per week for 4 weeks). Our research group has been working on MSC in other scenarios [29–32], especially in acute graft-versus-host disease [33, 34] with good results. Based on these previous studies, we hypothesize that a similar protocol might be able to change the course of ACLF. Another remarkable difference between the present study and the previous studies is the severity of liver disease in patients. As well as including noncirrhotic patients, some exclusion criteria, like history of variceal bleeding, recent infection, and severe renal failure [17], suggest patients were in better condition than those included in this study. All of our patients were cirrhotic and when randomized were in the intensive care unit for ACLF management. Six of the patients had ACLF Grade 2, and three had ACLF Grade 3, with high CP, MELD, and CLIF-C scores, evidencing a much more severely affected population than previously evaluated.

BM-MSC infusion was safe, without significant side effects, similar to previous studies [17–20]. In the follow-up, five patients died up to 4 days after the randomization process (one on the same day of the enrollment and another patient one day after randomization, both from the placebo group). Three patients in the intervention group died (all three having received only two doses of BM-MSC), without showing improvement in their clinical status or laboratory tests.

In terms of laboratory data, we examined patients closely from three days before the first infusion up to the seventh day in order to examine the acute effects of MSC on liver function and systemic inflammatory response. In general, there was a slight improvement seen in liver function in the MSC group compared to the placebo group. On the other hand, we were not able to show any improvement in inflammation. This is important, as the CANONIC trial [7] has demonstrated a worse inflammatory profile in patients with ACLF.

In terms of safety evaluation, this study has demonstrated that the MSC infusion is safe, when compared with previous reported trials [17–20], regardless of their source (bone marrow or umbilical cord), doses, or infusion sites. There were only two side effects observed (hypernatremia and gastric ulcer), which were presumed not to be associated with the treatment, as the low quantity of sodium in each infusion was not able to cause hypernatremia, and there is a lack of plausibility in the development of a gastric ulcer with MSC.

It is important to emphasize that the only patient in the intervention group who received the whole pre-established protocol five BM-MSC infusions has shown an improvement after the fourth dose, with a significant recovery in liver function; ACFL was resolved as well, enabling his discharge one week after the end of the study protocol, as shown in Figure 3. Although this study is underpowered to draw conclusions regarding mortality, aspects that may have reduced the benefits of the BM-MSC infusion in this study were related to the following flaws: (a) the low number of patients enrolled; (b) patients presenting an extremely severe disease (ACLF Grades 2 and 3), mean MELD of 38; and (c) the infusion protocol was not completed due to the high early mortality. We probably started the MSC therapy too late (when a systemic inflammatory response had already established), thus blocking the effects of the progenitor cells, as has been suggested by some other authors [35–37].

In conclusion, we evaluated a new protocol of infusion of BM-MSC and demonstrated the safety of this treatment in high grades of ACLF in cirrhotic patients. There was a definite improvement in liver function in one case, suggesting MSC therapy could be explored further, perhaps in less severe forms of ACLF, such as ACLF 1, and in a larger group of patients.

## Figures and Tables

**Table 1:** Clinical characteristics of patients at baseline.

Characteristics	Stem cell group (*n* = 4) *n* (%)	Placebo group (*n* = 5) *n* (%)
Age (years)	55.8 ± 12.8	53.4 ± 14.4
Gender		
Female	2 (50.0)	1 (20.0)
Male	2 (50.0)	4 (80.0)
Race		
White	4 (100.0)	4 (80.0)
Brown	0 (0.0)	1 (20.0)
Hypertension		
No	2 (50.0)	2 (40.0)
Yes	2 (50.0)	3 (60.0)
Insulin resistance		
No	4 (100.0)	3 (60.0)
Yes	0 (0.0)	2 (40.0)
Smoker		
No	3 (75.0)	5 (100.0)
Yes	1 (25.0)	0 (0.0)
Alcohol abuse		
No	4 (100.0)	4 (80.0)
Yes	0 (0.0)	1 (20.0)
Renal injury		
No	4 (100.0)	4 (80.0)
Yes	0 (0.0)	1 (20.0)
ACLF trigger		
Unknown	3 (75.0)	0 (0.0)
Non-SBP infection	0 (0.0)	2 (40.0)
SBP	1 (25.0)	1 (20.0)
Variceal bleeding	0 (0.0)	1 (20.0)
Surgery	0 (0.0)	1 (20.0)
Etiology		
Alcohol	1 (25.0)	2 (40.0)
Hepatitis C	1 (25.0)	3 (60.0)
NASH	1 (25.0)	0 (0.0)
Hepatitis B	1 (25.0)	0 (0.0)

^*∗*^Mean ± standard deviation. ^*∗∗*^*p* value from Chi-square test. ^*∗∗∗*^ACLF, acute-on-chronic liver failure; SBP, spontaneous bacterial peritonitis; NASH, nonalcoholic steatohepatitis.

**Table 2:** Biochemical and clinical scores of patients at baseline.

Characteristics	All (*n* = 9)	MSC group (*n* = 4)	Placebo group (*n* = 5)
Hb (g/dL)	9.5 ± 1.8	10.0 ± 1.3	9.0 ± 2.1
WBC (mm^3^)	9655.6 ± 599.3	7792.5 ± 5297.1	11146.0 ± 6668.9
PLT (×10^9^/mm^3^)	72.2 ± 58.1	53.5 ± 12.2	87.2 ± 77.5
TBili (mg/dL)	11.5 ± 10.3	12.4 ± 11.5	10.9 ± 10.6
INR	3.5 ± 1.5	3.9 ± 2.1	3.1 ± 0.8
sALB (g/dL)	2.7 ± 0.7	3.0 ± 1.0	2.4 ± 0.3
Cr (mg/dL)	2.8 ± 1.0	2.9 ± 0.8	2.7 ± 1.2
Na (mEq/L)	138.1 ± 4.9	140.5 ± 5.2	136.2 ± 4.1
AST (U/L)	102.1 ± 69.4	103 ± 54.7	101.4 ± 85.9
ALT (U/L)	95.9 ± 137.5	75.2 ± 42.9	112.4 ± 188.9
ALP (U/L)	114.9 ± 67.2	92.0 ± 54	133.2 ± 76.9
GGT (U/L)	106.0 ± 109.2	62.0 ± 4.2	123.6 ± 128.6
CRP (mg/dL)	54.0 ± 25.7	45.5 ± 23.6	60.8 ± 27.8
Child–Pugh	12.7 ± 1.2	12.2 ± 1.7	13.0 ± 0.7
MELD	38.4 ± 7.3	38.0 ± 11.3	38.8 ± 2.9
MELD-Na	37.8 ± 6.7	39.0 ± 10.2	36.8 ± 2.9
ACLF grade	2.3 ± 0.5	2.5 ± 0.6	2.2 ± 0.4
CLIF-C	50.7 ± 10.9	51.2 ± 6.2	50.2 ± 14.4

^*∗*^*p* value from Mann–Whitney *U* test. ^*∗∗*^MSC, mesenchymal stem cells; Hb, hemoglobin; WBC, white blood cell; PLT, platelet; TBili, total bilirubin; INR, international normalized ratio; sALB, serum albumin; Cr, creatinine; Na, sodium; AST, aspartate aminotransferase; ALT, alanine aminotransferase; ALP, alkaline phosphatase; GGT, gamma-glutamyltransferase; CRP, C-reactive protein; MELD, model for end-stage liver disease; ACLF, acute-on-chronic liver failure; CLIF-C, chronic liver failure consortium (https://www.clifresearch.com/ToolsCalculators.aspx).

## Data Availability

The data used to support the findings of this study are available from the corresponding author upon request.
